# Effect of sialidase inhibitors on a plaque community biofilm model

**DOI:** 10.1099/acmi.0.001143.v5

**Published:** 2026-06-22

**Authors:** Chandra Ramalingam, Katherine Ansbro, Jonathan Pratten, David Bradshaw, Graham P. Stafford

**Affiliations:** 1 School of Clinical Dentistry, University of Sheffield, Sheffield, S10 2TA, UK; 2 Oral health R&D, Haleon, Weybridge, K13 0DE, UK

**Keywords:** 16S sequencing, microbiome, MiSeq, ONT, sialidase

## Abstract

The oral microbiome is a diverse ecosystem that plays a critical role in health and disease and contains numerous bacterial species capable of metabolizing host-derived glycans, particularly sialic acids. Sialidase enzymes can be produced by both commensal and pathogenic bacteria influencing biofilm formation and host interactions. To investigate how sialidase activity might influence the oral microbiome, we conducted a series of *in vitro* polymicrobial biofilm experiments and assessed community composition using 16S rRNA sequencing. As a first step, we tested modified Oxford Nanopore Technology (ONT) primers using an in-house sequencing workflow and compared them to the standard Illumina MiSeq primers. Through *in silico* and *in vitro* assessments, we identified primer bias in the standard ONT 16S primers and designed human oral microbiome (HOM) modified primers (HOM_27F-YM/1492R-D) to improve taxonomic resolution, achieving results comparable to the gold-standard Illumina 16S primers particularly for key oral genera. These HOM-optimized primers had an overall lower error rate (3.4%) and generated community profiles that closely matched those produced by Illumina. We then used the same ONT workflow and modified 16S primers to evaluate the effects of the sialidase inhibitors oseltamivir and 2,3-dehydro-2-deoxy-*N*-acetylneuraminic acid on hydroxyapatite-coated minimum biofilm eradication concentration assay plate-derived plaque biofilms from a whole-plaque community model. Inhibitor-treated biofilms exhibited differences in relative abundance depending on the inhibitor combination used, with increased abundance of *Streptococcus* with oseltamivir alone and *Fusobacterium* with both inhibitors combined (Kruskal–Wallis cutoff=0.05, LDA>2). These findings demonstrate that ONT-based 16S sequencing with HOM-modified primers suggests that sialidase activity can modulate microbial community structure in plaque biofilms.

## Graphical abstract

MBEC-cultured 18-species and plaque biofilm models. Plaque biofilms were treated with sialidase inhibitors and assayed. Microbial community composition was profiled via Illumina MiSeq (18-species) and Nanopore sequencing, then visualised in R.

**Impact Statement**

Recent advances in 16S next-generation sequencing techniques have significantly improved our understanding of the human oral microbiome. 16S rRNA gene sequencing provides many advantages, such as cost-effectiveness and the ability to sequence in-house from low-biomass samples, as well as improved data analysis pipelines. In this study, we used modified human oral microbiome primers for use with Oxford Nanopore Technology sequencing, which was comparable to the standard Illumina MiSeq sequencing, to elucidate the effect of sialidase inhibitors on a plaque community model to understand their impact on the microbial community with time. Our work highlights the essential importance of metabolic or functional studies in uncovering mechanisms that govern microbial balance in complex biofilm communities while using specific primers and analysis pipelines for oral microbiome analysis.

## Data Summary

All genomic data generated and/or analysed during the current study are available from the European Nucleotide Archive under the study accessions PRJEB98716 and ERP181091. The accession numbers for the individual samples used in this study are provided in Table S3.

## Introduction

The oral microbiome is a complex community of micro-organisms, with over 700 bacterial taxa from 14 phyla identified [1,2]. The core oral microbiome comprises Gram-positive cocci like *Abiotrophia*, *Peptostreptococcus*, *Stomatococcus* or bacilli like *Actinomyces*, *Bifidobacterium*, *Corynebacterium*, *Eubacterium*, *Lactobacillus*, *Propionibacterium*, *Pseudoramibacter* and *Rothia* as well as Gram-negative cocci like *Moraxella*, *Neisseria* and *Veillonella* or anaerobes like *Campylobacter*, *Desulfobacter*, *Eikenella*, *Fusobacterium*, *Hemophilus*, *Leptotrichia*, *Prevotella*, *Selenomonas* and *Treponema* [1]. In nature, micro-organisms grow and aggregate as complex biofilm communities. Hence, *in vitro* biofilm community models offer a way of understanding the complexity of biofilms by mimicking naturally occurring biofilms within the human body, under controlled, laboratory conditions [3,4]. A commercially available model called the minimum biofilm eradication concentration (MBEC) assay (formerly known as Calgary Biofilm Device) currently exists that was developed in the 1990s [5]. The biofilm supporting surface consists of 96 vertically suspended pegs to grow biofilms, which can then be coated first in hydroxyapatite (HA) and then in a pellicle of the researcher’s choice to mimic the tooth surface and the acquired salivary pellicle [6].

In the case of the oral cavity, the salivary or subgingival gingival crevicular fluid pellicle coats all surfaces and is rich in glycoproteins, e.g. salivary agglutinin, proline-rich glycoproteins, mucins and secretory immunoglobulin A [7], and is important for early colonization of pioneer species of bacteria, such as streptococci [8]. Similar glycans are attached to almost all cell surfaces in the human body, conjugated to proteins and lipids (glycolipids) [9,10]. These glycans are usually composed of chains of several sugars in a variety of structural conformations from simple linear to complex multi-branched glycans [9]. They mediate cell-cell, cell-matrix, cell-molecule and host–microbiome interactions [10,11]. Commonly, human glycan chains are terminally decorated with the amino sugar sialic acid and attached to underlying sugars via glycosidic bonds [12]. In the human body, and in the mouth, sialic acid is harvested by bacterial sialidase enzymes [13,16]. Many commensal oral bacteria, such as *Streptococcus oralis* and *Streptococcus intermedius* [17], and several associated with periodontal disease, such as *Porphyromonas gingivalis*, *Tannerella forsythia* and *Treponema denticola*, can secrete sialidases to access this additional energy source and aid their adhesion [18,21]. The importance of oral sialidases in periodontitis was further highlighted by Gul *et al*. [22], who demonstrated higher levels of sialidases present in subgingival periodontal pockets. The role of bacterial sialidases in oral biofilm formation has also been studied [23,24]. For example, a *P. gingivalis* mutant lacking sialidase showed altered capsule synthesis and concomitant biofilm formation [18,25], while a *T. forsythia* mutant lacking a *nanH* sialidase deletion had reduced biofilm growth and host interactions [20,23, 26, 27].

Sialidase inhibitors like zanamivir and oseltamivir act by inhibiting viral neuraminidase [28]. Both are derived from the sialic acid analogue 2,3-dehydro-2-deoxy-N-acetylneuraminic acid (DANA), a sialic acid transition state mimic that binds the active site and inhibits activity [29]. Studies have also shown that these three inhibitors can mediate oral bacterial virulence and are able to reduce the activity of sialidases from *Treponema* [21], *P. gingivalis* [19,30, 31], some *Prevotella* species [13] and *T. forsythia* [15,20, 32]. However, the inhibitory concentrations for bacterial enzymes are typically in the micromolar to millimolar range vs. the nanomolar range for viral enzymes [33]. This abrogation of sialidases also affects human cell interactions and immune modulation for these pathogens [34,35]. Furthermore, this also holds for bacteria in polymicrobial infections and can affect host cell interactions even for non-sialidase-producing bacteria such as *Fusobacterium nucleatum* in a polymicrobial infection with *P. gingivalis* and *T. forsythia* [19,36].

Overall, the action of these inhibitors on oral bacterial sialidases in several orally important bacterial species suggests a potential strategy to intervene and inhibit bacterial growth and host interactions. This is particularly important in the context of oral health and disease, where the composition of the bacterial community on the tooth or in proximity to gingival tissues in subgingival biofilms is known to be strikingly different in health vs. conditions such as gingivitis and periodontitis [32,37,41]. To summarize many hundreds of articles, and at risk of over-simplification, in the case of periodontal diseases, a healthy oral microbiome shifts composition to a dysbiotic microbiome containing higher levels of proteolytic, anaerobic organisms such as those mentioned above. It is notable that several of these organisms also possess a range of glycosidase and sialidase enzymes, as well as catabolic genes [13,23, 42, 43], as do several early colonising streptococci [8,44, 45]. However, the potential role of glycan metabolism in the context of polymicrobial ‘natural’ oral microbial communities has not been examined to date.

One way to monitor microbial communities that has become more and more accessible in recent years is the use of 16S rRNA gene profiling [46,47] and has been used in the context of oral samples with several experimental setups [4,48,51]. This accessibility has been driven by improvements in sequencing technology [52,53] and improved DNA polymerases to allow amplification of a broad range of 16S genes. In parallel, the amount of reference sequences and human-niche specific databases for profiling has also expanded rapidly, with the expanded Human Oral Microbiome Database (eHOMD) now containing around 774 bacterial-species level taxa. Historically, the majority of studies utilize the accurate, high-throughput, but short-read based Illumina platform (150–250 bp typically). However, this has limitations in terms of its ability to only resolve a sequence down to genus level [54,56], when many studies would benefit from species-level knowledge.

The next generation of sequencing platforms now includes the chip-and-pore-based Oxford Nanopore Technology (ONT) [57,58]. ONT is an example of long-read sequencing technology and enables the analysis of the entire 16S gene and potentially a higher resolution of sequences to species level in many cases. However, studies have found that standard ONT primers can fail to identify important genera in the oral microbiome, such as *Pseudomonas* [59], *Prevotella*, *Porphyromonas* and *Faecalibacterium* [60]. Therefore, in this study, we tested original and modified primer sets and prototype pipelines for ONT-based 16S microbiome work, comparing the outputs to Illumina-based sequencing to develop the ability to utilize this technology for the monitoring of the oral microbiome. In this case, we initially tested ONT to monitor the composition of an 18-species inoculum mock community to understand its feasibility to monitor *in vitro* grown bacterial communities from subgingival plaque of periodontal patients to understand their response to sialidase inhibitors. The methods developed and exemplar data on the changes after drug treatment lay a platform for future studies that will be improved by updated ONT sequencing chemistries and base calling methods.

## Methods

### Strain selection and inoculation of an exemplar *in vitro* biofilm model

Eighteen bacterial strains were selected for a ‘mock’ *in vitro* community model and are listed in Table S1 (available in the online Supplementary Material). All strains except *T. denticola* were cultured on Fastidious Anaerobe Agar (Neogen, UK) supplemented with 5% oxalated horse blood (Thermo Scientific, USA), *T. forsythia* was supplemented with 1% N-acetylmuramic acid. *T. denticola* ATCC 35405 was grown anaerobically at 37 °C in both modified NOS medium (ATCC medium 1494) semi-solid agar and liquid broth.

Individual bacterial species from agar (1×10^7^) were inoculated into a liquid growth medium comprised of 10 mg ml^−1^ (1% w/v) bovine submaxillary gland mucin (Sigma-Aldrich, USA), 30% heat-inactivated human serum (Sigma-Aldrich, USA) and 50% modified fluid universal medium (mFUM) (Gmür and Guggenheim, 1983). The bovine submaxillary mucin was either autoclaved or filter-sterilized using 0.45 µm filters. The human serum was inactivated by heating at 65 °C for 30 min. This media (200 µl per well), containing bacterial communities, were then placed in the cells of MBEC 96-well HA-coated plates that had been preconditioned overnight with 100% heat-inactivated human serum (Sigma-Aldrich, USA) and incubated at 37 °C under anaerobic conditions and replaced with fresh growth medium every 3.5 days. The cells were harvested at days 14 and 21 from three pooled pegs by snapping off with sterile forceps, washed thrice and resuspended in 500 µl PBS. A hand-operated pestle motor mixer (VWR, USA) was used to carefully disrupt and harvest bacterial cells from the pegs for 2 min each.

### Collection and inoculation for the periodontal plaque model

Plaque samples were collected from ten patient volunteers who attended the Periodontology Clinic in the Charles Clifford Dental Hospital. Volunteers were included if they were over 18 years of age with a known diagnosis of chronic periodontitis. Patients who were receiving periodontal therapy or antibiotics during the previous 3 months, immunocompromised patients and non-English speakers were excluded from the study. Ethical approval was given by the National Research Ethics Service Committee Yorkshire and Humberside (study number 18/WM/0068, IRAS Ref No: 240510, STF Ref No. STH20225). All participants gave written informed consent prior to sampling. Subgingival plaque was scraped from the tooth surface with a fine periodontal curette and placed into sterile 2-ml Eppendorf tubes containing mFUM. Samples were immediately stored at −80 °C. To prepare the plaque biofilm inoculum, subgingival plaque samples were pooled and centrifuged at 10,000** *****g*** to obtain a cell pellet, which was resuspended in mFUM. Preconditioned MBEC pegs were suspended in 200 µl of this plaque inoculum and incubated as described above. The data were analysed from two independent plaque pools for oseltamivir only and three independent plaque pools for oseltamivir+DANA experiments (with three technical replicates each).

When sialidase inhibitors were included, on the same plaque community and conditions as above, they were used at concentrations of 100 uM oseltamivir and 250 uM DANA purchased from Carbosynth, UK. In all cases, media-only controls were included.

### PMA treatment and DNA extraction

The harvested biofilm cells were treated with propidium monoazide (PMA) dye with a 10-min photo-exposure (conditions determined during the study, not shown). DNA was extracted using the QIAamp DNA Mini Kit (QIAGEN, UK) according to the manufacturer’s instructions. Bacterial cells were lysed by 37 °C incubation for 1 h with 90 µl lysozyme (10 mg ml^−1^, prepared with Tris-EDTA buffer). The cells were further incubated with 24 µl proteinase K, 4.8 µl RNAse A (100 mg ml^−1^) and 300 µl kit Buffer AL at 56 °C for 30 min. After mixing and a brief centrifugation step, DNA was precipitated by adding 400 µl 100% ethanol. Washing steps with kit Buffer AW1 and AW2 were followed according to the kit manufacturer’s guidelines, and DNA was eluted in 100 µl kit Buffer AE and stored at −20 °C until quantitative analysis.

### 16S MiSeq sequencing

Following PMA treatment, amplicon pools were produced using the 27FYM (5′-AGAGTTTGATYMTGGCTCAG-3′) and 338 R-R (5′- TGCTGCCTCCCGTAGRAGT-3′) primers as well as the 10 nt primer-pad and MiSeq i5 and i7 adapters according to Illumina recommendations (https://support.illumina.com/documents/documentation/chemistry_documentation/16s/16s-metagenomic-library-prep-guide-15044223-b.pdf) (reaction conditions are summarized in Table S2). All experiments included control PCR reactions, i.e. no template control, a PMA-treated extraction negative control (reagents only), DNA extracted from a negative control peg (mFUM only) and a ZymoBIOMICS Microbial Community DNA Standard (Zymo Research, USA). Amplicon size and quality were visualized by agarose gel electrophoresis before purification using Agencourt AMPure XP beads (Beckman Coulter, USA), then quality controlled on an Agilent 2100 Bioanalyzer (DNA 1000 kit) and quantified using the Qubit^™^ dsDNA high-sensitivity (HS) Assay Kit (Thermo Fisher, USA). Finally, the amplicons were pooled in equal concentrations and paired-end sequenced using the Illumina MiSeq Nano platform using a 500-cycle kit (2×250 bp) V2 chemistry. Sequencing was performed by the DNA Sequencing Facility at the Department of Biochemistry, University of Cambridge, UK.

### 16S ONT sequencing

The full ~1,500 bp 16S rRNA gene was sequenced on either a MinION flow cell (R9.4.1) or a Flongle flow cell (R9.4.1) using the SQK-LSK109 kit. Amplicon libraries of PMA-treated DNA templates were prepared using either the 27F/1492R primer set included in the ONT kit (27F: 5′- AGAGTTTGATCCTGGCTCAG-3′, 1492R: 5′-CGGTTACCTTGTTACGACTT-3′) or using our modified human oral microbiome (HOM) versions 27F-YM/1492R-D barcoded primers (27F-YM: 5′-AGAGTTTGATYMTGGCTCAG-5′, 1492R-D: 5′- CGGDTACCTTGTTACGACTT-3′). The reaction volumes and PCR reaction conditions are summarized in Table S2. After PCR, the amplicons were quality checked as described above. The amplicons were then pooled together in equal concentrations and loaded onto either a Flongle flow cell or a MinION flow cell according to the manufacturer’s instructions (SQK-LSK109). MinION reads were basecalled in real-time using a MinIT device (Oxford Nanopore Technologies, UK), the Fastq sequencing data acquired and the barcode and adapter sequences trimmed and demultiplexed using Porechop (https://github.com/rrwick/Porechop/) and binned according to barcode sequence.

### Data analysis

Fastq files were first checked for quality using FastQC v0.11.8 (https://www.bioinformatics.babraham.ac.uk/projects/fastqc/). Demultiplexed sequence reads were then both analysed using the mothur software suite available at the time of the experiments: v1.42.3 [61,62]. For MiSeq sequence reads, sequences <300 bp and >350 bp in length were discarded, whereas for Nanopore sequence reads, sequences <1,470 and >1,540 bp were discarded. The remaining sequences were aligned to the silva 16S rRNA reference alignment [63]. Sequences with homopolymers of >8 bases in length were also discarded, as were duplicate sequences. The VSEARCH algorithm [64] was used to identify and remove chimeric sequences. MiSeq sequences were further denoised by clustering sequences that had ≤3 base differences together, whereas Nanopore sequences were clustered for those with ≤15 differences. After denoising, the sequences were classified using a Naïve Bayesian classifier [65] with the eHOMD 16S rRNA reference dataset v.15.1 [66]. Relative abundance plots were visualized using the package ggplot2 in RStudio v3.3.1 (http://www.rstudio.com/); abundances were normalized per sample so that the taxa sum equalled 100%. The *α*-diversity of the communities was calculated using the Simpson’s diversity index [67], and the samples were rarefied to an even depth and subsampled to a minimum depth to account for differences in sequencing depth. The *β*-diversity was measured using the Bray–Curtis index to calculate distance matrices [68], which were then visualized as box and whisker plots and principal coordinate analysis (PCoA) plots using the R packages ggplot2 and phyloseq [69], respectively. The microbiomeMarker package [70,71] was used for linear discriminant analysis effect size (LEfSe) analysis at the genus level, counts were normalized to counts per million and taxa with a Kruskal–Wallis *P*-value <0.05 and a linear discrimination analysis (LDA) effect size ≥2 were considered significantly enriched in each group.

## Results

### The sialidase inhibitor oseltamivir influences plaque microbiome communities

As a first step to compare the feasibility and reliability of using ONT to profile the human oral microbiome, we tested modified ONT primers and directly compared them with Illumina MiSeq technology on exemplar bacterial communities, as a preliminary pilot study. In all cases, we eliminated the influence of extracellular DNA (eDNA) by the use of PMA dye prior to DNA extraction. We assessed the potential for primer bias in silico by aligning the 16S genes of 29 representative oral species from the HOMD (v15.2) with the primer sequences for versions containing non-degenerate and degenerate 27F as well as the 3′ 16S gene end 1492R primers. As shown in Fig. S1A, the un-modified ONT primers contained several mismatches with eHOMD species. For 27F, the inclusion of the commonly used YM wobble positions restores perfect primer matches with several oral species, most notably *Aggregatibacter*, *Rothia* and *Scardovia* as well as commensal *Porphyromonas* species*.* In the case of the 1492R primer, almost all oral species contain mismatches at position 4 (5′−3′) that can be restored by inclusion of a degeneracy to base ‘D’ (G/A/T) (HOM_1492R-D (5′-CGG**D**TACCTTGTTACGACTT-3′) and potentially improve coverage of the oral microbiome. We also performed the same *in silico* analysis for the short-read primer 338R (R-R TGCTGCCTCCCGTAGRAGT) primer, showing no mismatches (data not shown).

Based on a literature review, the commonly used short-read primer set 27F-YM/338 R-R (27F-YM: 5′-AGAGTTTGATYMTGGCTCAG-3′, and 338 R-R: 5′-TGCTGCCTCCCGTAGRAGT-3′) was selected to amplify the ~310 bp V1–V2 hypervariable region of the 16S rRNA gene [72]. These primers contain degeneracies to ensure wide coverage of bacterial sequences across genera and have been optimized over many studies [73,74]. Importantly, the standard Nanopore 16S Barcoding Kit (2019) utilizes the forward primer 27F (5′-AGAGTTTGAT**CC**TGGCTCAG-3′) and the reverse primer 1492R (5′-CGGTTACCTTGTTACGACTT-3′), but the 27F primer lacks the two degenerate ‘wobble’ bases (highlighted in bold) that are present in the commonly used HOM_27F-YM forward primer (5′-AGAGTTTGAT**YM**TGGCTCAG-3′). To test whether the introduction of these degeneracies influenced the sequencing data, the 27F/1492R and HOM 27F-YM/1492R-D ONT primers were used on the ZymoBIOMICS^™^ mock community DNA standard (Fig. S1B) and an 18-species mixed DNA biofilm (Fig. S1C) before comparison to the same DNA samples using Illumina MiSeq (27F-YM and 338 R-R) primers. The relative abundance of the operational taxonomic units (OTUs) using HOM-modified primers was similar to that of MiSeq, as seen in Fig. S1C. For comparison between the ONT runs using different primers, we accounted for sequencing depth by normalizing abundances per sample and confirming similar total read counts across runs, thereby minimizing potential bias due to differences in sequencing depth. Overall, this preliminary study suggested the modified 27F-YM/1492R-D primer set used with the ONT MinION flow cell would produce a community composition equivalent to the widely used Illumina 27 F-338R primer sets and was, therefore, used for further work.

In addition to primer degeneracy bias, we next assessed the influence of flow cell type and base calling on calculated error rates vs. a standardized DNA community mix (ZymoBIOMICS^™^ Microbial Community DNA Standard). Please note here that, at the time of these experiments, we were using the most up-to-date chemistry, flow cells and base calling software available for ONT amplicon sequencing. Using the reference sample, the error rate was calculated from the total sum of mismatches to the reference sequences divided by the total sum of bases in the query sequences. Besides sequence read inaccuracy, it was important to calculate the error rate because if bacterial species-level OTUs were clustered at either 1.5% or 3% cut-off and the error rate was >3%, it would mean the OTUs could not be clustered to species level. We performed the same analysis using the version 9.4.1 Flongle cells, with similar results but an increase in error rate compared to the MinION flow cells (Fig. S1D). During the time of this study, the base calling software, Guppy, was also updated from v.3.2.6 to v.4.3.4, which improved the error rate overall. Fig. S1D shows that the error rate for the ONT primers was calculated at 5.7%, while using the HOM primers, this dropped to 4.6%, but with the updated version of Guppy, this further dropped to 3.4%. Flongle-sequenced libraries yielded lower total reads compared to MinION libraries; therefore, abundances were normalized per sample so that each sample sums to 100%, enabling direct comparison across runs.

**Fig. 1.**
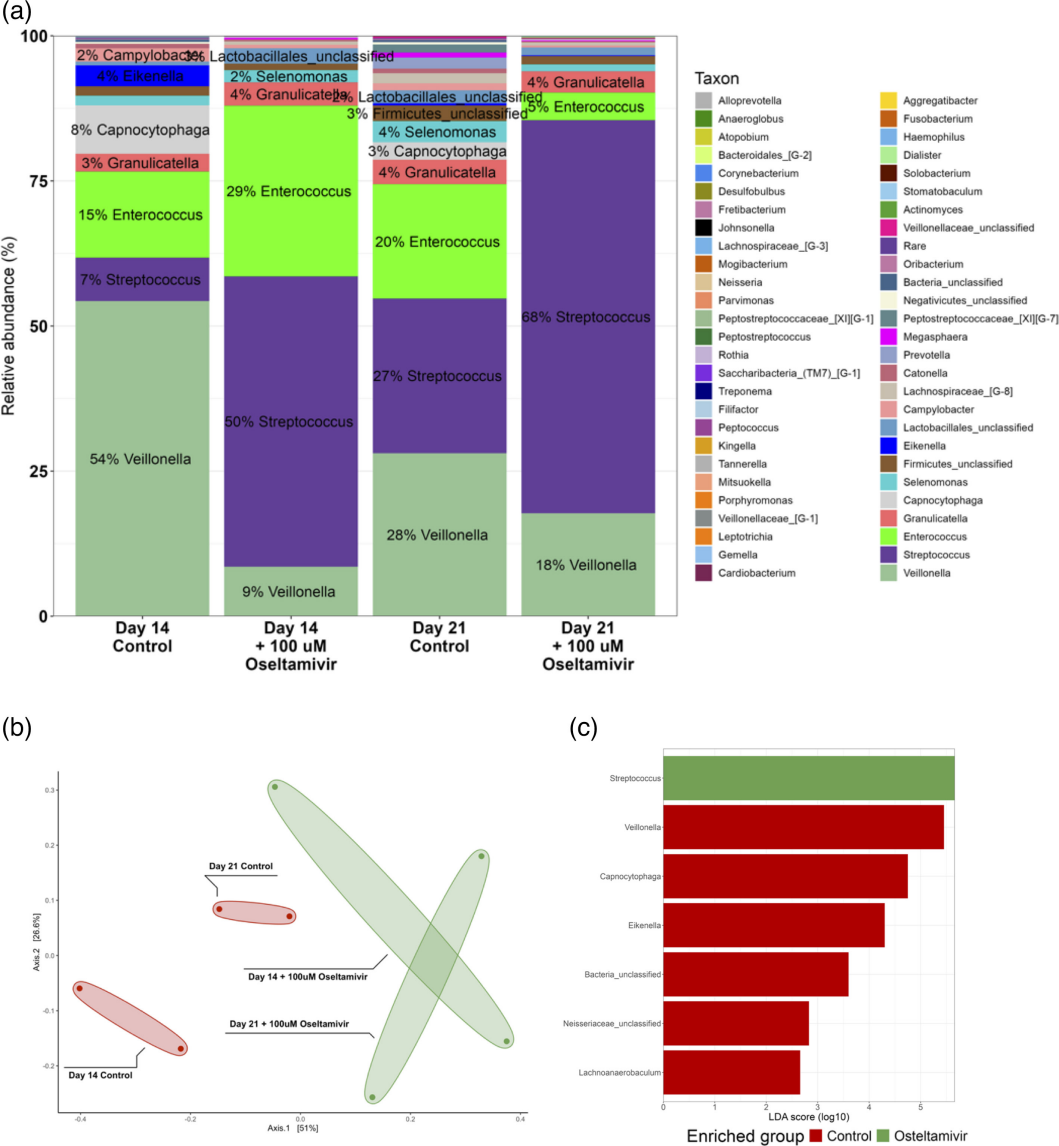
Community composition of plaque biofilms grown with and without 100 μM oseltamivir for 14 and 21 days. (a) The relative abundances of genus-level taxa that were >0.1% in at least one sample, (b) PCoA plots based on community *β*-diversity using the Bray–Curtis index and (c) LEfSe analysis showing significantly enriched (*P*<0.05; LDA score >2) genus-level taxa in oseltamivir-treated biofilms (combined days 14 and 21). The data were analysed from two independent plaque pools (with three technical replicates).

We then tested if the ONT methodology was applicable for relatively high-throughput experimental analyses (5 days from extraction to data) and using a treatment condition. In this case, we used the MBEC plates described and pooled samples of subgingival plaque (loosely based on the methodology developed by our collaborator William Wade [6]). We aimed to investigate the influence of inhibiting sialidase activity on bacterial communities. In this first experiment, we used the U.S. Food and Drug Administration (FDA)-approved anti-flu agent oseltamivir (Tamiflu), using a concentration of 100 μM, which we had established inhibits the sialidase of oral species both individually and from similar biofilm communities (Fig. S3 [20,32]). As shown in Fig. 1a (control samples), the microbial community established using this method of plaque inoculation from 10-pooled patient plaque samples with feeding every 3.5 days resulted in a diverse *in vitro* community of oral microbes that contained a range of genera, including *Veillonella*, *Streptococcus*, *Enterococcus*, *Campylobacter* and *Capnocytophaga*, representing an oral microbial community as had been found in previous work [6].

These communities (control samples) also showed varied *β*-diversity (Fig. 1) between 14 and 21 days, while the oseltamivir-treated biofilms at day 14 and 21 clustered together, indicating similar *β*-diversity when compared to the controls, which clustered separately. LEfSe analysis (Fig. 1) across both timepoints indicated that *Streptococcus* was the most enriched genus in oseltamivir-treated biofilms (Kruskal–Wallis cutoff=0.05, LDA >2), while *Veillonella*, *Capnocytophaga* (a sialidase-containing bacterium) and *Eikenella* were predominantly enriched in the control samples (i.e. depleted during sialidase inhibition). To note, these results were statistically significant when the results from both days 14 and 21 were assessed together. In addition, *α*-diversity analysis using the Simpson index showed decreased *α*-diversity in oseltamivir-treated biofilms at days 14 and 21 (Fig. S2A); however, this was not statistically significant. Despite these limitations, the data taken together suggested that the presence of 100 µM oseltamivir caused an overall change in the composition of the plaque communities.

### Influence of combined sialidase inhibitors on plaque microbiome communities

Based on our biochemical assays of sialidase activity of *in vitro* plaque biofilms (Fig. S3), the two most effective of commonly utilized sialidase inhibitors were oseltamivir (half-maximal inhibitory concentration (IC_50_=0.78 µM) and DANA (2.06 µM), while siastatin B (11.40 µM) and zanamivir (1.611 mM) had much higher IC_50_. We then tested a combination of these using equimolar quantities on whole biofilms (Fig. S4), with successful inhibition of activity. In addition, we also tested inhibition of sialidase (100 × IC_50 _: 250 µM and 100 µM for DANA and oseltamivir, respectively) on whole cells of a range of oral bacteria present in subgingival plaque, showing that different species had different susceptibilities (Fig. S5). As a result, and as we could not test all 700+ species in the oral microbiome, and because both drugs were FDA approved, we tested oseltamivir and DANA in combination on community profiles with independent samples from the oseltamivir-only experiment. Furthermore, we note that neither drug is antimicrobial in our hands (data not shown).

**Fig. 2.**
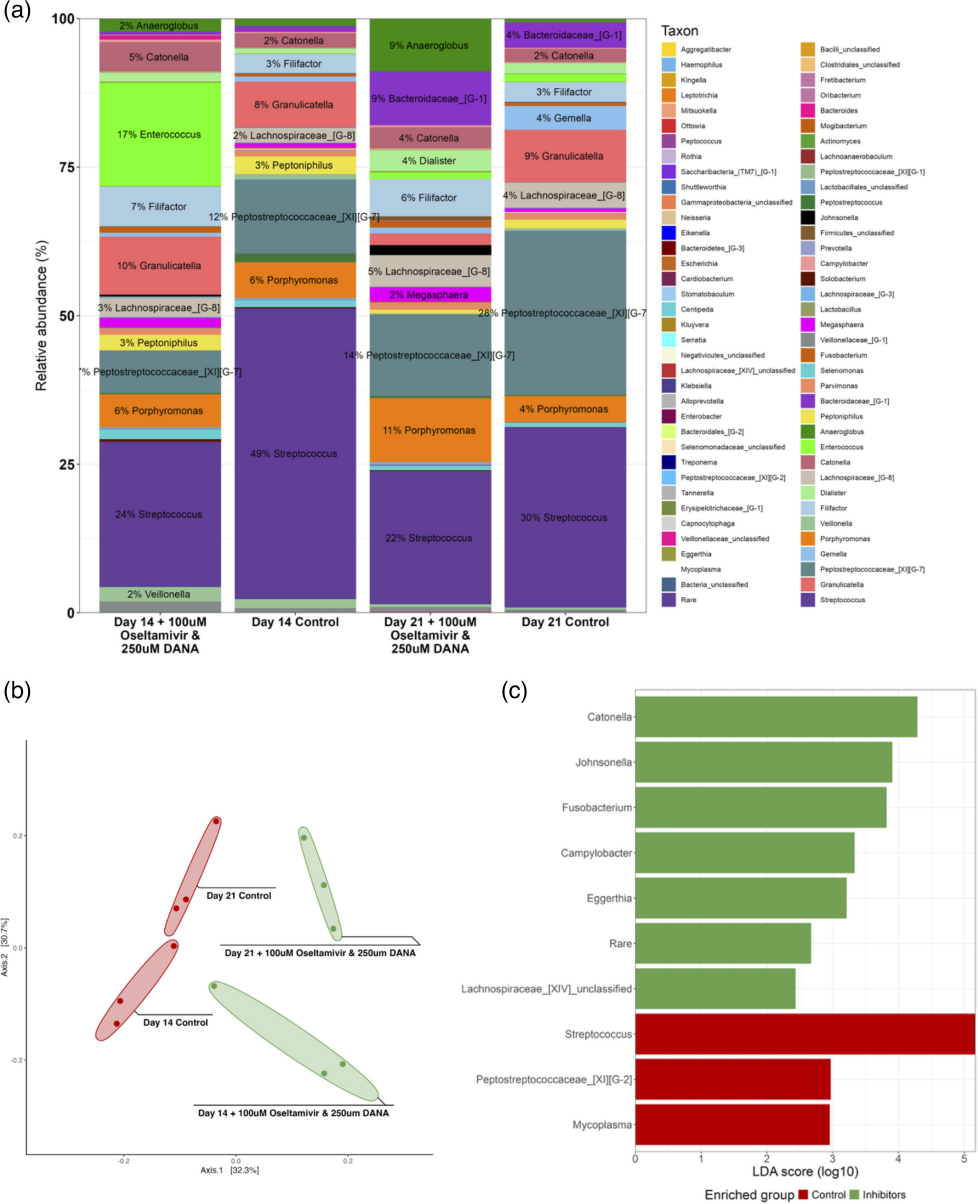
Community composition of plaque biofilms grown with and without 100 μM oseltamivir and 250 µM DANA for 14 and 21 days. (a) The relative abundances of genus-level taxa that were >0.1% in at least one sample, (b) PCoA plots based on community *β*-diversity using the Bray–Curtis index and (c) LEfSe analysis showing significantly enriched (*P*<0.05; LDA score >2) genus-level taxa in oseltamivir- and DANA-treated biofilms (combined days 14 and 21). The data were analysed from three independent plaque pools (with three technical replicates).

Next, biofilms from three separate pooled plaque biofilm samples (three MBEC pegs each) were created for each condition. The samples were analysed for their relative abundance using ONT sequencing on days 14 and 21 (Fig. 2a). Across all the biofilm communities, irrespective of the age of the biofilms and whether the inhibitors were added, *Streptococcus* species dominated with mean relative abundances of 49% and 30% at days 14 and 21, respectively. Generally, across the biofilms grown, the six most dominant genus-level taxa apart from *Streptococcus* were *Peptostreptococcaceae_[XI][G-7]*, *Granulicatella*, *Veillonella*, *Porphyromonas*, *Peptoniphilus* and *Filifactor*. Comparison between the biofilms grown for 14 and 21 days, irrespective of the presence or absence of inhibitors, showed an overall reduction in the relative abundances of the taxa *Veillonella* and an increase in *Streptococcus* and *Peptostreptococcaceae_[XI][G-7]* and a decrease in *Veillonella*, *Filifactor* and *Granulicatella* at day 14. At day 21, however, there was an increase in the relative abundance of *Streptococcus*, *Peptostreptococcaceae_[XI][G-7]*, *Granulicatella* and *Gemella* and a decrease in *Porphyromonas*, *Filifactor* and *Catonella*, when compared to the control samples. This indicated that the age of the biofilms appeared to influence the plaque community compositions. Here, *α*-diversity analysis (Fig. S2B) of the samples by the Simpson diversity index demonstrated that *α*-diversity increased with the age of biofilm, although this was not statistically significant. The *β*-diversity showed distinct clustering (Fig. 2) between the control samples and the inhibitor-treated biofilms at days 14 and 21; however, the controls as well as the inhibitor-treated biofilms clustered closer to each other. LEfSe analysis (Fig. 2) of the combined 14- and 21-day data indicated that *Catonella*, *Johnsonella* and *Fusobacterium* were the most enriched genera in drug-treated biofilms (Kruskal–Wallis cutoff=0.05, LDA>2), while *Streptococcus* dominated the control biofilm.

## Discussion

This study aimed to study the effect of sialidase inhibition on an *in vitro* oral polymicrobial biofilm using a set of modified long-read ONT primers improved for oral microbiome 16S profiling. We demonstrate the feasibility of this method for relatively high-throughput (5 days), in-house microbiome analysis. We chose a standalone ONT approach because studies show that the kit chemistry provides the high accuracy (>99.9% consensus) required for reliable genus-level identification without the need for short-read polishing [75,76]. By sequencing the full ~1,500 bp 16S rRNA gene (V1–V9) with modified primers, we improved taxonomic resolution that short-read or hybrid approaches often miss in complex oral samples [77,79]. Furthermore, using a single-platform workflow minimizes cross-platform biases, allows for quicker, more cost-effective and real-time analysis. By modifying the standard ONT primer sets to include degenerate bases, we were able to reduce primer bias and improve taxonomic resolution, producing community profiles that matched those generated using Illumina MiSeq short-read sequencing. Standard ONT primers introduced mismatches across multiple oral taxa, particularly within genera such as *Aggregatibacter*, *Rothia* and *Scardovia*. This likely contributed to the underrepresentation of these groups for non-degenerate primers. Several studies have demonstrated that the use of degenerate primers offers advantages over non-degenerate primers, including detection of increased microbial diversity and low-abundance bacteria [80], as well as species-specific amplification [52]. We also assessed the influence of ONT flow cell type and base calling software on sequencing accuracy. Our data demonstrate that although earlier versions of ONT base calling and Flongle flow cells had elevated error rates (>4%), updated Guppy software combined with HOM-primers reduced error rates below 4%, which allows for more reliable species-level OTU clustering [57]. This, coupled with oral-specific databases such as the eHOMD, allows for better processing of oral microbiome data.

Although several bioinformatic pipelines are available for analysis, such as Kraken2 (which is more suitable for metagenomic classification) [81], Minimap2 (which was originally designed for aligning genomic DNA to a reference only) [82] and Emu (which was released fairly recently) [83], we have adapted the mothur [62] pipeline to be suitable for ONT data. At the time of this study, mothur was deemed to be the most relevant pipeline for OTU clustering at the genus level. In the past few years, several updates have been released by ONT to improve flow cell and kit chemistry, library preparation methods, base calling accuracy and post-sequencing correction tools like Nanopolish and Medaka [84]. These findings support the growing feasibility of using ONT sequencing for precise taxonomic profiling in oral microbiome research, particularly when primer selection and base calling tools are optimized.

Since our study was focused on 16S rRNA sequencing, host DNA depletion was not taken into consideration since this is of more relevance for shotgun metagenomics rather than 16S sequencing [85,86]. Studies have also shown several limitations of including this additional step in sample prep, such as damage to bacterial cells depending upon their cell wall fragility [87] and the removal of all cell-free microbial DNA [88], which can lead to depletion and underrepresentation of important microbial DNA. Furthermore, host DNA is even less of an issue with subgingival plaque samples collected with curettes, which are then grown in laboratory conditions and hence contain even less potential for human DNA contamination [89].

Our application of the ONT platform to study the effects of sialidase inhibition using oseltamivir revealed significant alterations in overall microbial community structure. Oseltamivir-treated biofilms showed altered diversity and increased dominance of *Streptococcus*, alongside decreases in genera such as *Veillonella*, *Capnocytophaga* and *Eikenella*. The enrichment of *Streptococcus* is consistent with its competitive advantage under conditions of reduced glycoprotein degradation, potentially due to lower levels of liberated sialic acid, a nutrient source for many commensals and pathobionts. However, without species-specific data, these data are hard to interpret [8,17, 44, 45, 90]. Meanwhile, *Capnocytophaga* is a sialidase producer that is known to require sialidase to access glycoproteins [91], while there are reports of *Eikenella* species requiring sialidase removal of glycans to adhere to glycoproteins on human cells [92,93] and *Veillonella* requiring a sialic acid binding adhesin for interaction with *Streptococcus* [94,95]. Hence, these data might correlate with the inhibition of sialidase, observed using enzyme assays (Fig. S5), which could be confirmed by measurement of free sialic levels.

In our dual drug experiments, we observed changes in overall community structure and differential proportions of some species after drug treatment, e.g. *Streptococcus*, but again, given a lack of knowledge of which species were altered, it is hard to interpret further. These changes in community structure are reminiscent of altered expression of sialidase and its influence on the gut [9,11, 14] and vaginal [13] microbiomes and suggest a potential role for sialidase activity in the dynamics of the oral microbiome also.

Despite these promising findings, our results must be interpreted in light of several limitations. Firstly, while sequencing accuracy has improved, ONT still lags behind Illumina in terms of raw base-call fidelity [96,98], and results may vary depending on the specific software and flow cell used. Secondly, although PMA treatment was used to eliminate eDNA, we cannot fully exclude differential amplification biases [99,100]. Thirdly, the experimental models, while well-controlled, involved a limited number of replicates, which may limit the generalizability of the findings. Nonetheless, the consistency of taxonomic trends across independent experiments supports the robustness of our findings and suggests that glycan utilization via sialidases is an important part of the nutritional landscape of the oral microbiome.

## Conclusion

We have demonstrated that long-read ONT sequencing, optimized using modified primers and updated base calling software, provides a feasible and accurate approach for real-time 16S rRNA oral microbiome profiling that closely matches Illumina-based community profiling. Our findings highlight the importance of primer design and bioinformatic pipeline adaptation for improving taxonomic resolution and sequencing accuracy. Application of this platform to investigate sialidase inhibition revealed notable shifts in microbial community structure and synergistic effects of dual inhibitors on sialidase activity across species. While ONT sequencing still faces challenges in raw accuracy and amplification biases, the reproducible microbial trends observed support its growing utility for high-throughput, in-house oral microbiome research.

## Supplementary material

10.1099/acmi.0.001143.v5 Supplementary Material 1.

10.1099/acmi.0.001143.v5 Supplementary Material 2.

## Abbreviations

DANA: 2,3-dehydro-2-deoxy-N-acetylneuraminic acid
eDNA: extracellular DNA
eHOMD: expanded Human Oral Microbiome Database
FDA: Food and Drug Administration
HA: hydroxyapatite
HOM: human oral microbiome
LDA: linear discrimination analysis
LEfSe: linear discriminant analysis effect size
MBEC: minimum biofilm eradication concentration
mFUM: modified fluid universal medium
ONT: Oxford Nanopore Technology
OTUs: operational taxonomic units
PCoA: principal coordinate analysis
PMA: propidium monoazide
