# Supplementary material for: Effect of sialidase inhibitors on a plaque community biofilm model
Source: Access Microbiol. 2026 Jun 22;8(6):001143.v5. doi: 10.1099/acmi.0.001143.v5 (PMC13286422; doi:10.1099/acmi.0.001143.v5)
Supplement: Supplementary Material 1. [file acmi-8-01143-s001.pdf]

SUPPLEMENTARY INFORMATION

ADDITIONAL METHODS

4-Methylumbelliferyl N-acetyl-a-D-neuraminic acid (MUNANA) assay for detecting bacterial sialidase activity

Sialidase activity was quantified using MUNANA assays by observing the cleavage of the substrate 4-Methylumbelliferyl N-acetyl-a-D-neuraminic acid (MUNANA) to produce Neu5Ac and the fluorochrome 4-Methylumbelliferone (4-MU) (Potier et al., 1979). MUNANA assays were performed for each of the 18 bacteria present in the mock community model. Bacterial colonies were suspended in 1 ml PBS pH 7.4 at a final optical density OD600 = 0.5 and standard curves for 4-MU were created by 1:1 serial dilutions of 1 mM 4-MU (final concentrations 4-MU at 0.0014-0.0909 mM). In total reaction volumes of 110 µl in black flat-bottomed 96 well polystyrene plates (Greiner, UK), the following were then added: 10 µl 4-MU or bacterial suspension; 90 µl PBS and 10 µl 2mM MUNANA. A blank control was also included, and the reactions were incubated at 37 °C for 4 h. Reactions were then quenched by the addition of 50 µl 100 mM Na2CO3 buffer pH 10.5 and the fluorescence measured at excitation 355 nm and emission at 430 nm using a Tecan Infinite M200 microplate reader. Concentrations of 4-MU were then extrapolated using the 4- MU standard curve.

Detection and inhibition of sialidase activity in biofilm whole cell and supernatant

Sialidase activity was quantified following the protocol described previously using the MUNANA assay, with the exception of resuspending the frozen biofilm whole cell pellets at an optical density OD600 = 0.5. In place of whole cell samples, supernatant (i.e. “spent” growth medium from biofilm models) collected were diluted 1 in 10 prior to use. The sialidase inhibitors zanamivir (GlaxoSmithKline UK), oseltamivir (Carbosynth, UK), siastatin B (Carbosynth, UK) and N-Acetyl-2-3-dehydro-2-deoxyneuraminic acid DANA (Carbosynth, UK) at 10 mM were serially diluted 1:2 into reaction mixtures and tested in the MUNANA assays at final inhibitor concentrations of 0-0.909 mM. MUNANA reactions were stopped

and read 4 h after incubation. Sialidase activity of the samples in reactions where an inhibitor was present was expressed as the percentage change in fluorescence, when compared to fluorescence in reactions where inhibitors were absent. This is denoted by the formula:

$$\frac{\text{Fluorescence at 0.0005 to 0.909 mM inhibitor}}{\text{Fluorescence at 0 mM inhibitor}} \times 100 = \text{change in fluorescence (\%)}$$

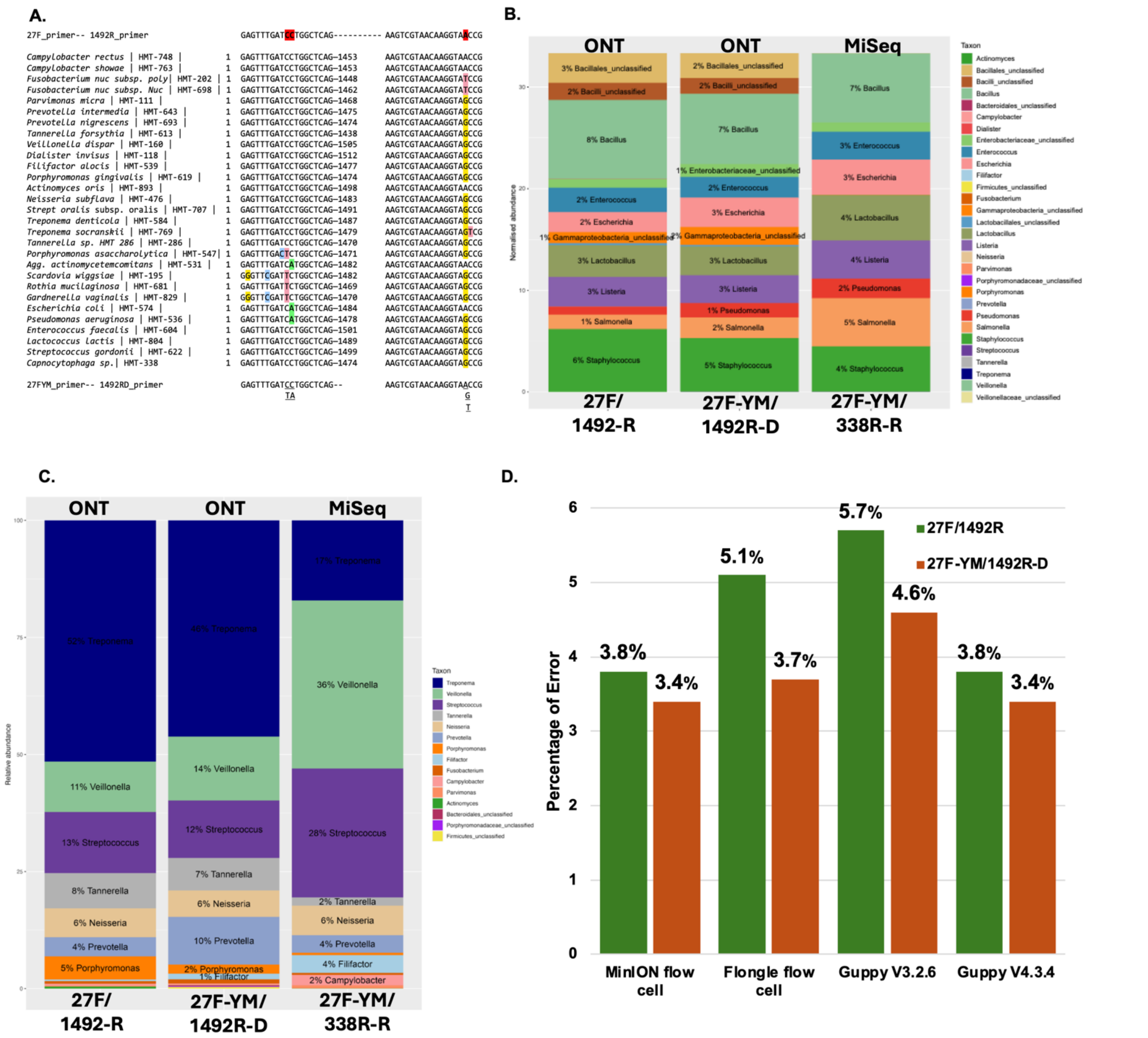

Figure S1: Comparison of 16S rRNA ONT primers A) Modified (HOM) forward 27F and reverse 1492R primers (wobble bases highlighted in red) B) Relative abundance of genus-level taxa with Zymobiomics mock community C) Relative abundances of genus-level taxa in the 18-species *in vitro* mock community model using original and HOM ONT primers and standard Illumina MiSeq primers D) Percentage of error when using modified 16S rRNA ONT primers with different ONT flow cells and different versions of Guppy basecalling software with Zymobiomics mock community

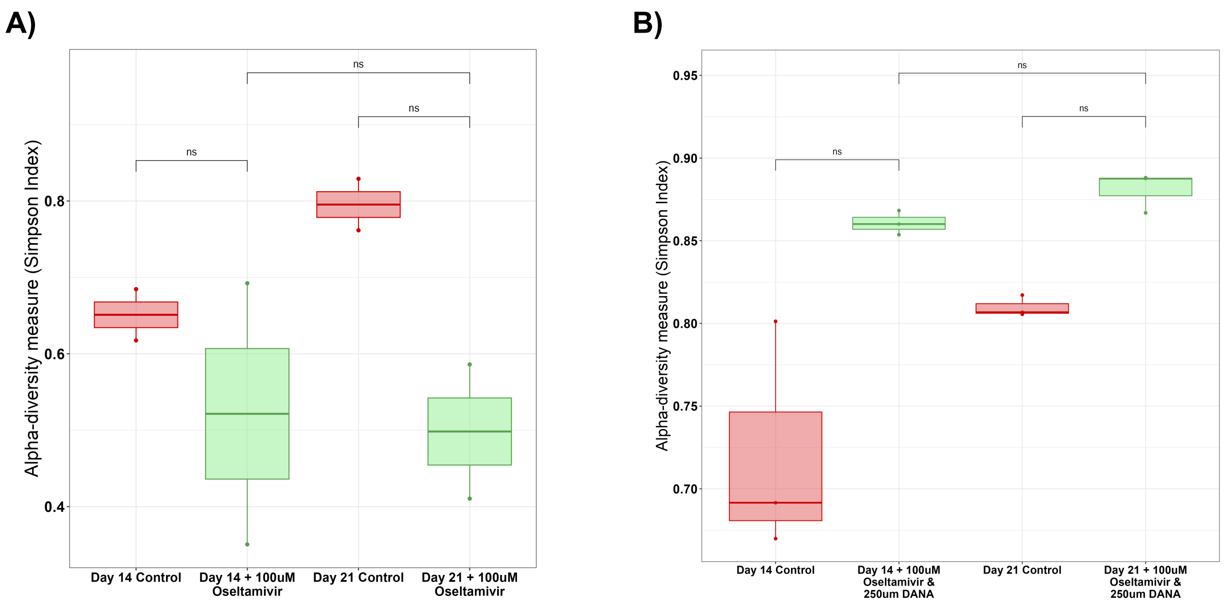

Figure S2: Comparison of α-diversity metrics in day 14 and 21 inhibitor-treated biofilms. A) Boxplots depicting the Simpson's α-diversity index values in 100 uM Oseltamivir-only treated biofilms at day 14 and 21 and B) Boxplots depicting the Simpson's α-diversity index values in 100 uM Oseltamivir and 250 uM DANA treated biofilms at day 14 and 21. ns = non-significant.

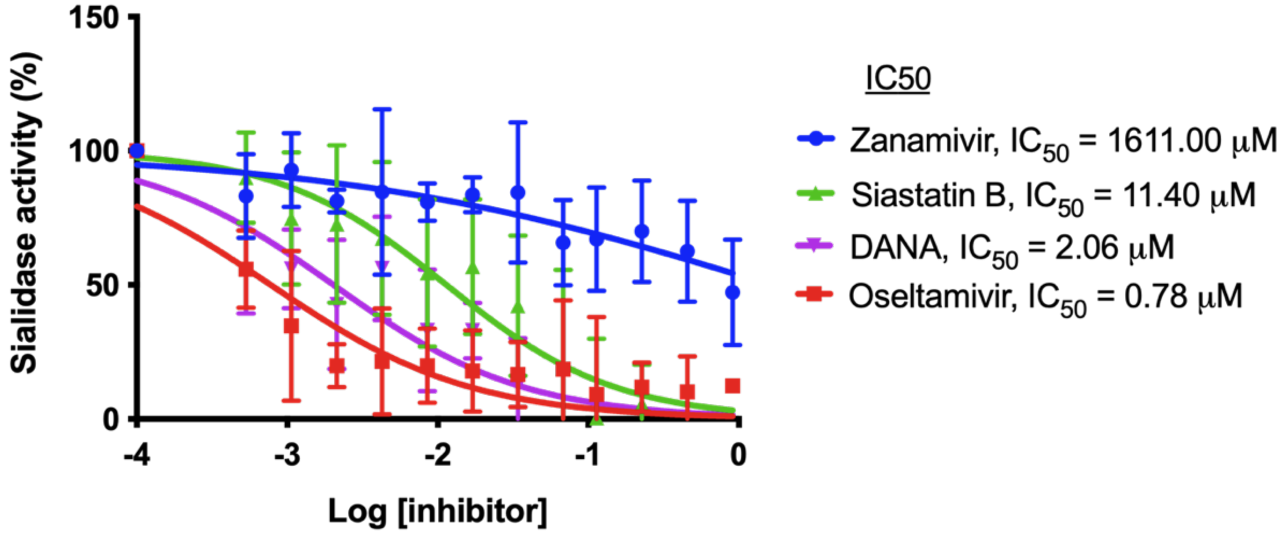

Figure S3: Half-maximal inhibitory concentration (IC_{50}) values measured for four sialidase inhibitors. IC_{50} curve depicting the Log[inhibitor] against percentage of sialidase

activity (%) relative to absent of each of the inhibitors: Zanamivir, Siastatin B, N-Acetyl-2,3-dehydro-2-deoxyneuraminic acid (DANA) and Oseltamivir. IC_{50} values were expressed as μM whereby a 50 % reduction in fluorescence was measured for reactions where an inhibitor was present when compared to reactions in the absence of an inhibitor. Data represent the mean of three biological repeats and error bars calculated from standard error of mean (SEM).

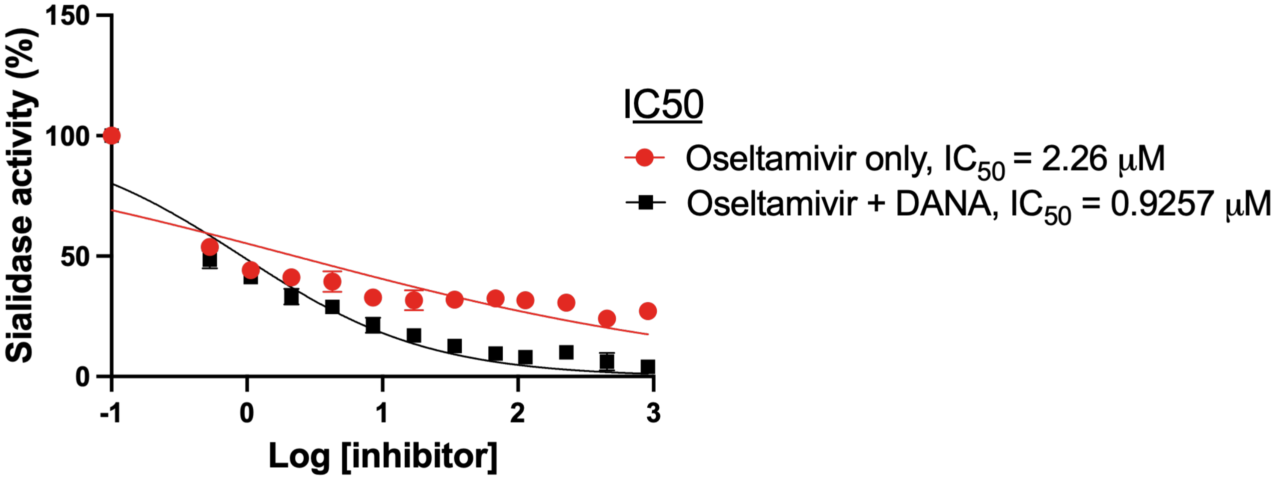

Figure S4: Half-maximal inhibitory concentration (IC_{50}) values measured for Oseltamivir and Oseltamivir combined with DANA. IC_{50} curve depicting the Log[inhibitor] against percentage of sialidase activity (%) relative to absent of either Oseltamivir only, or Oseltamivir in combination with DANA in biofilm supernatant collected during multispecies biofilm growth. IC_{50} values are expressed as μM whereby a 50 % reduction in fluorescence is measured for reactions where an inhibitor is present when compared to reactions with absence of inhibitor. Data represent the mean of three biological repeats and error bars calculated from standard error of mean (SEM).

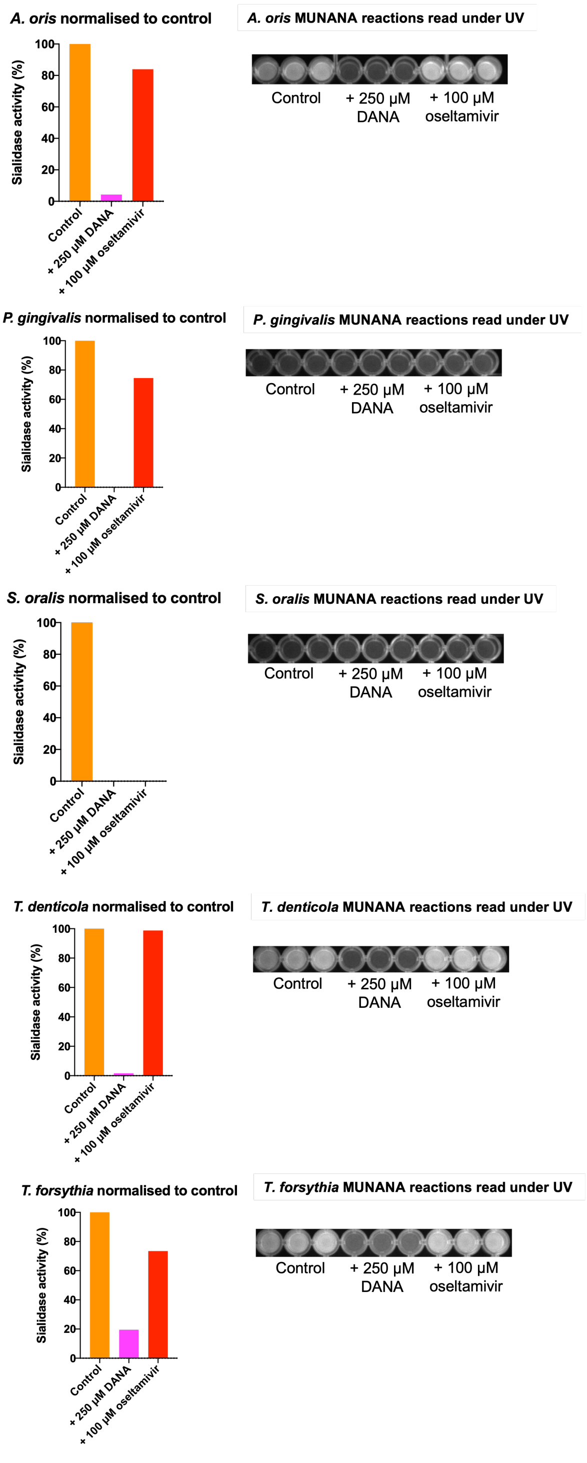

Figure S5: MUNANA assay measurements on sialidase-positive bacteria treated with sialidase inhibitors. Bacterial species which were previously confirmed to exhibit sialidase activity were tested separately in a MUNANA assay without the presence of any inhibitors (control), with the addition of 250 µM DANA and with the addition of 100 µM oseltamivir. Fluorescence was most measured for the bacterial species A. oris, T.denticola and T.

forsythia and this was also confirmed by visualising the reactions under UV light as depicted by the brightness of the control wells when compared to the control wells of P. gingivalis and S. oralis. The addition of 250 μM DANA also caused the greatest reduction in fluorescence and in sialidase activity, with 100 μM oseltamivir demonstrating partial effect. C. rectus was included to act as a negative control and therefore its sialidase activity for the addition of inhibitors was not compared against the control reactions.
